# Extradural Motor Cortex Stimulation might improve episodic and working memory in patients with Parkinson’s disease

**DOI:** 10.1038/s41531-020-00129-8

**Published:** 2020-09-28

**Authors:** Carla Piano, Marco Ciavarro, Francesco Bove, Daniela Di Giuda, Fabrizio Cocciolillo, Anna Rita Bentivoglio, Beatrice Cioni, Tommaso Tufo, Paolo Calabresi, Antonio Daniele

**Affiliations:** 1 Neurology Unit, IRCCS Fondazione Policlinico Universitario A. Gemelli, Rome, Italy; 2 IRCCS Neuromed, Pozzilli, Italy; 3 Department of Neuroscience, Università Cattolica del Sacro Cuore, Rome, Italy; 4 Nuclear Medicine Unit, IRCCS Fondazione Policlinico Universitario A. Gemelli, Rome, Italy; 5 Department of Radiological and Hematological Sciences, Università Cattolica del Sacro Cuore, Rome, Italy; 6 Neurosurgery Unit, IRCCS Fondazione Policlinico Universitario A. Gemelli, Rome, Italy

**Keywords:** Neurophysiology, Neuroscience

## Abstract

Electric Extradural Motor Cortex Stimulation (EMCS) is a neurosurgical procedure suggested for treatment of patients with advanced Parkinson’s disease (PD). We report two PD patients treated by EMCS, who experienced worsening of motor symptoms and cognition 5 years after surgery, when EMCS batteries became discharged. One month after EMCS restoration, they experienced a subjective improvement of motor symptoms and cognition. Neuropsychological assessments were carried out before replacement of batteries (off-EMCS condition) and 6 months afterward (on-EMCS condition). As compared to off-EMCS condition, in on-EMCS condition both patients showed an improvement on tasks of verbal episodic memory and backward spatial short-term/working memory task, and a decline on tasks of selective visual attention and forward spatial short-term memory. These findings suggest that in PD patients EMCS may induce slight beneficial effects on motor symptoms and cognitive processes involved in verbal episodic memory and in active manipulation of information stored in working memory.

## Introduction

Electric chronic Extradural Motor Cortex Stimulation (EMCS) is a minimally invasive neurosurgical procedure requiring implantation of extradural electrodes. EMCS has been suggested for treating patients with Parkinson’s disease (PD) who refuse deep brain stimulation (DBS) or are not eligible for DBS^1,2^, due to contraindications to such more invasive neurosurgical procedure (age >70 years, dementia, marked brain atrophy, or diffuse chronic ischemic encephalopathy on brain neuroimaging). Preliminary observations in PD patients treated by EMCS suggest that electrical stimulation of the primary motor cortex (PMC) may modulate neural activity in the basal ganglia (BG), by activating cortico-BG projections^1,3^, resulting in slight improvement of Parkinsonian motor symptoms, especially axial motor symptoms^4,5^. The effects of EMCS on cognitive functioning in patients with PD remain to be clarified^5,6^. In PD patients treated by bilateral EMCS, comparisons between preoperative and postoperative cognitive performance on neuropsychological tasks did not detect any significant overall cognitive decline at a 6-month^5^ and 1-year postoperative follow-up^5,6^. One study showed at 3-month postoperative assessment a significant but transient decline of performance (increased execution time) only on the Stroop interference test, a task assessing response inhibition and sensitive to frontal lobe dysfunction^5^. By contrast, a statistical trend toward a post-operative improvement was detected in the same study at 6-month and 12-month postoperative assessments on a task of verbal episodic long-term memory (LTM), which might have been influenced by a practice effect^5^.

In a case of pure akinesia with gait freezing, a variant of progressive supranuclear palsy (PSP), bilateral EMCS improved motor symptoms (speech and gait), and cognitive performance on language (sublexical and lexical-semantic) tasks^7^.

We report here two PD patients with rigid-akinetic phenotype, who underwent EMCS, with bilateral implantation over PMC.

Approximately 5 and a half years after surgery, both patients gradually started to experience a deterioration of motor symptoms and cognition, noticed also by their relatives. When visiting patients, we found that EMCS batteries were discharged. One month after the batteries were replaced, patients experienced a subjective improvement of motor symptoms and cognition. In order to investigate possible changes in cognitive performance, apparently related to battery status, patients underwent an extensive neuropsychological assessment before (off-EMCS) and 6 months after (on-EMCS) replacement of batteries.

## Results

### Case reports

Patient 1 is a man with a 11-year history of PD, who started to show at the age of 59 years Parkinsonian motor symptoms (rigidity, bradykinesia) and later developed “on–off” fluctuations and peak-dose dyskinesias. He underwent bilateral EMCS implantation at the age of 70 years, since DBS was contraindicated by lacunar state in the BG, detected by magnetic resonance imaging. Patient 2 is a man with a 9-year history of PD, who started to show at the age of 57 years Parkinsonian motor symptoms (rigidity, bradykinesia) and later developed postural instability. He underwent bilateral EMCS implantation at the age of 66 years, since he refused DBS and showed prominent axial Parkinsonian symptoms (freezing of gait, postural instability), with poor response to dopaminergic drugs.

After the EMCS procedure, mild beneficial effects on motor symptoms (particularly, akinesia and freezing of gait) and a reduced frequency of falls were seen in both patients. Over time, motor scores assessed by Unified Parkinson’s Disease Rating Scale (UPDRS), part III slowly deteriorated in both patients (Table 1), with an increase of few points (<5) at 60-month postoperative follow-up. Dopaminergic therapy was reduced over the years following EMCS, indicating a reduced need for pharmacological management of motor symptoms. These findings suggest that EMCS might have slight long-standing beneficial effects on PD motor symptoms, which is a favorable outcome in such progressive neurodegenerative disorder.

**Table 1:** Motor assessment, Levodopa Equivalent Daily dose, and anti-parkinsonian drugs.

		Patient 1			Patient 2			
	Baseline	60-month follow-up	66 months off-EMCS	72 months on-EMCS	Baseline	60-month follow-up	66 months off-EMCS	72 months on-EMCS
UPDRS off-med	34	36	39	36	21	26	39	35
UPDRS on-med	16	23	23	23	16	25	27	27
LEDD	1257	979	1335	1219	1450	1175	700	850
Pharmacological treatment	–Levodopa/ entacapone 825 mg/die –Ropinirole 8 mg/die	–Levodopa/ entacapone 300 mg/die –Amantadine 300 mg –Rotigotine 6 mg	–Levodopa/ entacapone 575 mg/die –Levodopa 150 mg/die –Safinamide 100 mg/die –Amantadine 200 mg –Rotigotine 4 mg	–Levodopa/ entacapone 300 mg/die –Levodopa 400 mg/die –Safinamide 100 mg/die –Amantadine 200 mg –Rotigotine 4 mg	–Levodopa 1250 mg/die –Ropinirole 10 mg/die	–Levodopa 1075 mg/die –Rasagiline 1 mg/die	–Levodopa 600 mg/die –Rasagiline 1 mg/die	–Levodopa 750 mg/die –Rasagiline 1 mg/die

Motor assessment by means of the Unified Parkinson’s Disease Rating Scale (UPDRS) part III, Levodopa Equivalent Daily dose (LEDD) and pharmacological treatment with anti-parkinsonian drugs.

Some months after the 60-month postoperative routine follow-up, Patient 1 reported a deterioration of motor symptoms (increased “wearing off” periods, severe rigidity) and cognition (impaired concentration, memory loss), while Patient 2 complained about severe akinesia, gait and balance impairment, memory loss, impaired concentration. Both patients required an unscheduled neurological assessment. On that occasion, the pulse generator of the battery hand unexpectedly lost charge in Patient 1 (battery was at “end of life”), while in Patient 2 generator was switched off, since battery was out of charge entirely. At that time (66 and 68 months after EMCS, respectively), patients underwent motor and cognitive assessments in off-EMCS and showed a deterioration of UPDRS-III motor scores in off-medication condition (Table 1), as compared to 60-month assessment. After 3 days, batteries were replaced in both patients. About 1 month after again switching on the EMCS device, patients reported a subjective improvement of motor symptoms and cognition via reviews undertaken by telephone. Six months after switching on the device, patients were reassessed and, as compared with off-EMCS, motor scores in off-medication condition improved (Table 1).

As to dosage of anti-parkinsonian drugs (Table 1), in Patient 1 Levodopa-Equivalent Daily Dose (LEDD) was 1257 at baseline and was reduced to 979 at 60-month follow-up. After deterioration of motor symptoms was reported by Patient 1 when battery-life was at the end, an increase of LEDD to 1335 was deemed necessary. Six months after restarting EMCS, motor symptoms improved and LEDD was reduced to 1219. In Patient 2, LEDD was 1.250 at baseline and was reduced to 1.175 at 60-month follow-up. Although motor symptoms of Patient 2 worsened when battery was out of charge, LEDD was reduced to 600 at 68-month assessment, due to episodes of orthostatic hypotension. At 74-month assessment, LEDD was increased to 850, in an attempt to improve motor symptoms.

Table 2 reports the results obtained on neuropsychological tests by both PD patients on three conditions (preoperative baseline, off-EMCS condition, on-EMCS condition). Comparisons were made between on-EMCS versus off-EMCS conditions and between the off-EMCS versus baseline conditions.

**Table 2:** Cognitive assessment.

Test (Score Range)	Patient 1								Patient 2							
	Baseline		off-EMCS		on-EMCS		% Change		Baseline		off-EMCS		on-EMCS%		%Change	
	CS	ES	CS	ES	CS	ES	off-EMCS vs. baseline	on-EMCS vs. off-EMCS	CS	ES	CS	ES	CS	ES	off-EMCS vs. baseline	on-EMCS vs. off-EMCS
MMSE (0–30)	29.5	–	29	–	30	–	*−2%*	**+1%**	28.5	–	27.9	–	27.9	–	*−2%*	–
RAVLT Immediate Recall (0–75)	39	3	31.9	1	40.3	3	*−18%*	**+26%**	43.7	4	45.7	4	53.2	4	**+5%**	**+16%**
RAVLT Delayed Recall (0–15)	10.3	4	5.6	1	11.9	4	*−46%*	**+112%**	8.7	3	8.7	4	9.9	4	–	**+13%**
Digit Span backward	4	3	3.4	2	4.4	4	*−15%*	**+29%**	7	4	5.2	4	5.2	4	*−26%*	–
Corsi Span Backward	4	3	3.2	1	4.3	3	*−20%*	**+34%**	4.2	3	3.5	2	4.5	3	*−16%*	**+29%**
RPM ‘47	34	4	20.6	1	28.6	4	*−39%*	**+38%**	31.6	4	29.6	4	26.8	3	*−6%*	*−10%*
Semantic verbal fluency	20	4	14.4	3	17.4	4	*−28%*	**+21%**	16.8	4	16.7	4	13.8	2	*−1%*	*−17%*
Letter verbal fuency	25.2	3	27.9	3	22.9	2	**+10%**	*−18%*	33.1	4	20.3	1	20.5	1	*−39%*	**+1%**
Digit span forward	5	4	5.4	4	4.4	1	**+8%**	*−19%*	6.1	4	6.3	4	7.3	4	**+4%**	+16%
Corsi span forward	4	1	5.4	3	4.4	2	**+35%**	*−18%*	4	1	5.2	4	4.3	1	**+29%**	*−18%*
MFCT: accuracy	1	–	0.9	–	0.8	–	*−10%*	*−18%*	1	–	0.9	–	0.9	–	*−10%*	*−5%*
MFCT: false alarms	0	4	0	4	2	1	*–*	*–*	0	4	0	4	1	3	*–*	*–*
MFCT: time	40	4	58.7	4	264.4	0	*+47%*	*+350%*	41.2	4	68.8	4	72.8	4	*+67%*	*+6%*
Stroop (time)	11.5	4	9	4	6.5	4	**−22%**	**−37%**	16.7	4	6.4	4	14.9	4	**−61%**	*+134%*
Stroop (errors)	0	4	2.5	2	1.6	2	–	**−37%**	0	4	0	4	2.8	1	–	–
Copy of drawings	9.5	3	8.6	2	9.6	3	*−9%*	**+12%**	8.8	2	9	2	9	2	**+3%**	–
Copy of drawings with landmark	69.3	4	58	0	67	2	*−16%*	**+15%**	69.8	4	68.3	3	68.3	3	*−2%*	–

For each variable, the percentage of changes (off-EMCS vs baseline and on-EMCS vs off-EMCS) shown in the table were obtained from each individual patient (Italics: decline = poorer performance; Bold: improvement = better clinical condition).

*MMSE* Mini-Mental State Examination, *RPM* ‘*47* Raven’s Progressive Matrices ‘47, *RAVLT* Rey’s Auditory Verbal Learning Test*, CS* corrected scores, *ES* equivalent scores.

At preoperative baseline (about 5 and a half years before batteries became discharged), both patients showed performance in the normal range on all neuropsychological tasks, with scores in lower normal range only on a spatial short-term/working memory (WM) task (Corsi Block-Tapping Test forward).

As compared to off-EMCS condition, in on-EMCS condition (about 6 months after replacement of batteries of the EMCS implant), both patients showed an improved performance on the backward spatial short-term/WM task (Corsi span backward: +34% in Patient 1; +29% in Patient 2) and the episodic verbal memory task (Rey’s Auditory Verbal Learning Test, RAVLT immediate recall: +26% in Patient 1; +16% in Patient 2; RAVLT delayed recall: +112% in Patient 1; +13% in Patient 2). Furthermore, in Patient 1 we observed an improved performance on the backward verbal short-memory task (Digit span backward: +29%) and the nonverbal abstract reasoning task (Raven’s Progressive Matrices, RPM ’47: +38%).

By contrast, in on-EMCS condition as compared to off-EMCS condition, we observed in both patients a decline of performance on the forward spatial short-memory test (Corsi Spatial forward: −18% in both patients) and the selective visual attention task (Multiple Feature Target Cancellation, MFTC), particularly as regards time of execution in Patient 1 (time of execution: −350%) and, in both patients to a lesser extent, as regards accuracy (Patient 1, −18%; Patient 2, −5%). In addition, in Patient 1 a decline was detected on tasks of verbal forward short-term memory (Digit span forward: −19%) and phonological fluency (Letter verbal fluency: −18%).

In off-EMCS condition, as compared to preoperative baseline, we observed in both patients an improved performance on the Stroop interference test as to time of execution (Patient 1: -22%; Patient 2: −61% in), on forward Corsi spatial short-memory task (Patient 1: +35%; Patient 2: +29%), and a very slight improvement on a forward verbal short-memory task /Digit span (Patient 1: +8% in; Patient 2: +4%).

By contrast, in off-EMCS condition as compared to preoperative baseline, neuropsychological assessment showed in both patients a decline in cognitive performance on tasks of backward verbal (Digit span in Patient 1: −15%; Patient 2: −26%) and spatial Corsi short-memory (Patient 1: −20%; Patient 2: −16%), on the nonverbal abstract reasoning task/RPM 47 (Patient 1: −39%; Patient 2: −6%) and on the selective visual attention task (MFTC), as regards both accuracy (−10% in Patient 1; −10% in Patient 2) and time of execution (+47% in Patient 1; +67% in Patient 2). Moreover, only in Patient 1 there was a decline in performance in off-EMCS condition on two subtests of the episodic verbal memory test (RAVLT immediate recall: -18%; RAVLT delayed recall: −46%), on two subtests of constructional apraxia (Copy of drawings: −9%; Copy of drawings with landmarks: −16%), on the semantic verbal fluency task (−28%). Furthermore, only in Patient 2 there was a decline in performance in off-EMCS condition on the phonological verbal fluency task (Letter verbal Fluency: −39%).

## Discussion

In both our PD patients treated by EMCS, there was a subjective deterioration of motor symptoms and cognitive functioning when batteries of pulse generator had lost charge (66 and 68 months after surgery, respectively). One month after battery replacement, a subjective improvement of motor symptoms and cognition was reported by both patients. In agreement with such subjective cognitive improvement associated with EMCS restoration, 6 months after replacement of batteries an objective cognitive improvement was detected on neuropsychological tasks assessing backward spatial short-term/WM and episodic verbal memory.

Since patterns of cognitive changes observed across different assessments in our patients is quite complex, we will mainly focus on the findings which seem to us more remarkable, by taking into account not only percent of changes but also changes in equivalent scores across assessments.

In Patient 1, who showed the most marked cognitive improvement after battery replacement, as compared to the off-EMCS condition, we observed in on-EMCS condition a remarkable improvement of performance on tasks assessing episodic verbal memory (immediate and delayed recall of RAVLT, which involve retrieval processes of verbal information stored in LTM) and nonverbal abstract reasoning (RPM ’47). Furthermore, we observed a slight improvement on tasks assessing semantic verbal fluency and response inhibition (Stroop interference test), and on tasks of short-term/WM requiring active manipulation of information stored in WM (backward digit and spatial span). In Patient 2, as compared to the off-EMCS condition, we observed in on-EMCS condition a slight improvement on a task of short-term/WM (backward spatial span) and a remarkable improvement of performance on an episodic verbal memory subtest (immediate recall of RAVLT). The improvement on a task of episodic verbal memory (immediate recall subtest of RAVLT test), which cannot be explained in our two patients by a practice effect, is at least partially consistent with the results of a previous study carried out in 9 PD patients treated with EMCS^5^, showing a statistical trend towards an improved performance on the same task at 6- and 12-month postoperative assessments.

Despite some differences in such patterns of improvement in on-EMCS condition across the two PD patients, we speculate that restoration of chronic electric stimulation in these subjects may have induced beneficial effects on activity of neural circuits involving prefrontal and parietal cortical areas, which play a critical role in retrieval of information stored in LTM and in processes of active manipulation of information in WM. The improved performance in on-EMCS condition on a task of spatial short-term/WM is in agreement with experimental study on an animal model of PD, in which optogenetic stimulation of secondary motor cortex (combined with L-DOPA treatment) resulted in improvement of WM^8^. It was hypothesized^9^ that EMCS might modulate the activity of the hyperdirect pathway, namely of glutamatergic projections from subthalamic nucleus, which receives inputs from PMC and prefrontal cortex^10^. It could be speculated that modulation of the hyperdirect pathway might play some role in beneficial effects on cognitive tasks in which prefrontal cortical areas play a critical role.

By contrast, the deterioration of cognitive performance in on-EMCS condition (as compared to off-EMCS condition) on other tests (tasks of selective visual attention and forward spatial short-memory in both patients, tasks of verbal forward short-term memory and phonological fluency in Patient 1) seems to us of difficult interpretation. Possibly, restoration of chronic electric stimulation in these subjects may have detrimental effects mainly on activity of neural circuits which play a critical role in cognitive processes underlying selective visual attention task and storage of visuo-spatial information in short-term memory.

The improvement of cognitive performance in off-EMCS condition as compared to preoperative baseline (5 and 1/2 years earlier) in both patients on some cognitive tasks (forward spatial short-memory task, reduced execution time in the Stroop Interference test) is of interest, but of not easy interpretation. We speculate that, despite being accidentally interrupted for some time before off-EMCS assessment, EMCS might have induced, over a period of more than 5 years, phenomena of neural plasticity in cortical-subcortical circuits underlying cognitive processes, such as response inhibition. This latter hypothesis, however, is not easy to reconcile with the observation of an increased execution time on the Stroop Interference test in PD patients treated by EMCS^5^, at least in early phases (namely, at 3-month postoperative assessment).

In experimental rodent models of PD, high frequency stimulation of glutamatergic corticostriatal inputs may induce opposite forms of synaptic plasticity, such as long-term potentiation (LTP) and long-term depression (LTD). Both corticostriatal LTP and LTD depend on functional state of postsynaptic neurons, integrity or denervation of nigral dopaminergic inputs, and concomitant administration of L-DOPA^11,12^. Moreover, in PD experimental models motor and cognitive functions can be differentially affected by distinct changes in corticostriatal synaptic plasticity induced by high-frequency stimulation^13–15^. Thus, such complex synaptic changes may account for beneficial and detrimental effects observed in our patients following EMCS.

The deterioration of cognitive performance in off-EMCS condition as compared to preoperative baseline (about 5 years before) on other cognitive tasks (mainly on tasks assessing verbal and spatial short-memory, nonverbal abstract reasoning, selective visual attention) could possibly be due to detrimental effects of disease progression on some cognitive processes, despite phenomena of neural plasticity induced by EMCS.

EMCS does require at least 1 month to induce, through phenomena of neural plasticity, beneficial effects in patients with various neurological disorders (chronic pain, stroke, PD)^5,16–19^, Accordingly, beneficial effects of EMCS were subjectively reported by our patients about 1 month after restarting of EMCS and persisted up to 6 months. Thus, although a placebo effect could be hypothesized to explain beneficial effects observed after restarting of EMCS, such hypothesis seems unlikely. It remains to be clarified whether in our PD patients an improvement on some cognitive tasks might partially reflect beneficial effects of EMCS on motor symptoms.

A limitation of the present report is the lack of assessments in a sham condition or in a blinded condition. However, a sham assessment is difficult in patients treated by EMCS, since EMCS requires at least 1 month to induce clinical effects, as mentioned above. Moreover, since our patients were not aware that batteries had lost charge, assessments in OFF-EMCS could be considered close to a single-blinded evaluation.

Further studies on a larger sample size and with long-term follow-up (which should foresee a matched control group and metabolic/functional imaging) are certainly needed in order to confirm the present preliminary findings observed in our two PD patients.

The main findings observed in the two patients reported here, assessed before and after restoration of EMCS, suggest that in PD patients EMCS might induce beneficial effects on motor symptoms and cognitive processes involved in verbal episodic LTM (as already suggested by a previous study^5^) and in active manipulation of information stored in WM. Since EMCS induced beneficial effects on linguistic functions in a patient affected by PSP^7^, it is possible that EMCS might induce beneficial effects on different cognitive processes in distinct movement disorders.

## Methods

### Study design

**Fig. 1:**
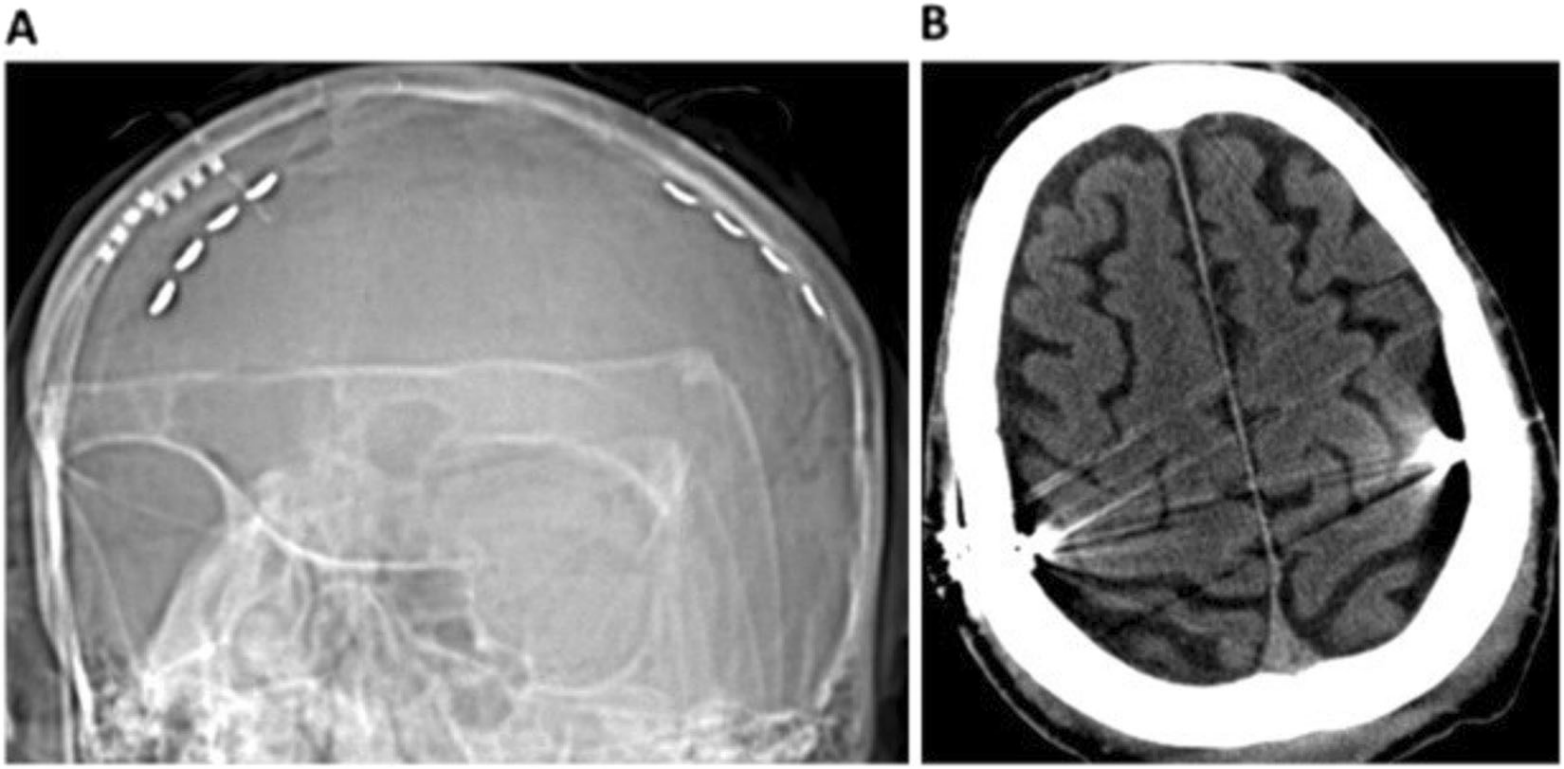
Position of the quadripolar electrode strip over the motor cortex. The figure shows the quadripolar electrode strip in Patient 1 oriented along the craniocaudal axis of the precentral gyrus (**a**), placed bilaterally over the motor cortex (**b**).

In both patients, a quadripolar electrode strip (Resume, Medtronic) was placed extradurally over the PMC of both cerebral hemispheres (Fig.1), using neuronavigation, intraoperative neurophysiology, and the phase reversal technique to identify the central sulcus^20^. The stimulation was continuously delivered through the two most distant contacts of the electrode paddle under a bipolar setting (parameters: 3-3.5 V, 120 μs, 80 Hz).

Both patients underwent a preoperative cognitive assessment (1 week before electrode implantation) and periodic postoperative follow-up cognitive assessment (at 6, 12, 36, 60 months post-surgery, not reported here), by means of an extensive neuropsychological test battery^5^, assessing various cognitive domains (short-term/working memory, LTM, attention, praxic abilities, language, abstract reasoning, and executive/frontal cognitive functions). The neuropsychological battery included the Mini-Mental State Examination (MMSE), tasks of spatial (forward and backward Corsi Block-Tapping Test) and verbal (forward and backward Digit Span) short-term/WM, tasks of verbal episodic LTM (RAVLT) with parallel versions to minimize practice effects, tasks of selective visual attention (MFTC), nonverbal abstract reasoning (RPM ‘47), phonological and semantic verbal fluency, response inhibition (Stroop interference test), and constructional praxis (Copy of drawings and Copy of drawings with landmarks).

Moreover, additional postoperative neuropsychological assessments (reported in detail here) were carried out before (off-EMCS condition, when the battery status was very low) and 6 months after the substitution of batteries of pulse generators (on-EMCS condition). All neuropsychological assessments were carried out in on-medication conditions. The ethics committee of the Catholic University of Sacred Hearth approved the study (#400-A763), and patients provide written informed consent.

## Supplementary information

Reporting-summary

## Data Availability

All data generated or analyzed during this study are included in this published article.
